# Bevacizumab for metachronous metastatic colorectal cancer: a reflection of community based practice

**DOI:** 10.1186/s12885-016-2158-8

**Published:** 2016-02-16

**Authors:** L. G. E. M. Razenberg, Y. R. B. M. van Gestel, I. H. J. T. de Hingh, O. J. L. Loosveld, G. Vreugdenhil, L. V. Beerepoot, G. J. Creemers, V. E. P. P. Lemmens

**Affiliations:** Department of Oncology, Catharina Hospital, Michelangelolaan 2, 5623 EJ Eindhoven, The Netherlands; Netherlands Comprehensive Cancer Organization (IKNL), Godebaldkwartier 419, 3511 DT Utrecht, The Netherlands; Department of Surgery, Catharina Hospital, Michelangelolaan 2, 5623 EJ Eindhoven, The Netherlands; Department of Oncology, Amphia Hospital, Langendijk 75, 4819 EV Breda, The Netherlands; Department of Oncology, Maxima Medical Centre, De Run 4600, 5504 DB Veldhoven, The Netherlands; Department of Oncology, Elisabeth-TweeSteden Hospital, Hilvarenbeekse Weg 60, 5022 GC Tilburg, The Netherlands; Department of Public Health, Erasmus MC University Medical Centre, Wytemaweg 8, 3015 CN Rotterdam, The Netherlands

**Keywords:** Colorectal cancer, Metachronous metastases, Palliative, Chemotherapy, Targeted therapy, Bevacizumab

## Abstract

**Background:**

Although the efficacy of bevacizumab has been established in patients with metastatic colorectal cancer (mCRC), population-based studies are needed to gain insight into the actual implementation of bevacizumab in daily practice. Since these studies are lacking for patients with metachronous metastases, the aim of this study is to evaluate the current role of bevacizumab in the treatment of metachronous metastases of CRC.

**Methods:**

Data on the use of bevacizumab as palliative treatment of metachronous metastases were collected for patients diagnosed with M0 CRC between 2003 and 2008 in the Eindhoven Cancer Registry (*n* = 361). Median follow up was 5.3 years.

**Results:**

One hundred eighty-five patients received bevacizumab in addition to first-line palliative chemotherapy (51 %), ranging from 36 % to 80 % between hospitals of diagnosis (*p* < 0.0001). Combined cytostatic regimens (CAPOX/FOLFOX in 97 %) were prescribed in the majority of patients (63 %) and were associated with a higher odds for additional treatment with bevacizumab than single-agent cytostatic regimens (OR 9.9, 95 % CI 5.51–18.00). Median overall survival (OS) rates were 21.6 and 13.9 months with and without the addition of bevacizumab to palliative systemic treatment respectively (*p* < 0.0001). The addition of bevacizumab to palliative chemotherapy was associated with a reduced hazard ratio for death (HR 0.6, 95 % CI 0.45–0.73) after adjustment for patient- and tumor characteristics and the prescribed chemotherapeutic regimen.

**Conclusion:**

Bevacizumab is adopted as a therapeutic option for metachronous metastasized CRC mainly in addition to first-line oxaliplatin-based regimens, and was associated with a reduced risk of death. The presence of inter-hospital differences in the prescription of bevacizumab reflected important differences in attitude and policies in clinical practice. Ongoing efforts should be made to further define the position of targeted agents in the treatment of metastatic colorectal cancer.

## Background

Metastatic disease is a common manifestation in patients with advanced colorectal cancer (CRC). Approximately one fifth of patients presents with metastasized disease at diagnosis [1–3] and 20 % of patients with initial M0 disease develops metachronous metastases [4].

Fluorouracil based palliative chemotherapy has been the mainstay of treatment for many years. Over the past decade, the systemic treatment of metastatic CRC (mCRC) has changed considerably. The availability of the cytostatic drugs irinotecan and oxaliplatin has improved the prognosis of mCRC patients [5]. Moreover, advances in the understanding of molecular oncology have served for the development of targeted agents such as the anti-vascular endothelial growth factor blocking agent (VEGF-a) bevacizumab. Although the efficacy of bevacizumab has been established in patients with mCRC [6], the role of bevacizumab in clinical practice remains a topic of debate. Population-based data are useful in reflecting community based practice. To date, no such population-based figures of patients with metachronous metastases are available. Therefore the aim of this study is to provide population-based data on the use and effect on overall survival of bevacizumab in the palliative treatment of metachronous metastasized CRC in the Netherlands.

## Methods

### Patients and data

Data from the population-based Netherlands Cancer Registry (NCR), more specifically from the Eindhoven area, were used. The Eindhoven Cancer Registry (ECR) collects data of all patients with newly diagnosed cancer in a large part of the Southern Netherlands. The ECR covers an area of approximately 2.4 million inhabitants, six pathology departments, ten hospitals and two radiotherapy institutions. Patient and tumor characteristics are collected from medical records by specially trained registry staff after notification by pathologists and medical registration offices, resulting in high quality of the data. The completeness of cancer registration is estimated to exceed 95 %. In the ECR, primary tumors are classified according to the TNM classification of Malignant Tumors by the international Union Against Cancer (UICC), 7^th^ edition [7]. Additional data were retrospectively collected on metachronous metastases for patients diagnosed between 2003 and 2008 with stage I-III CRC. Hospitals were asked to participate in the study by giving permission to use their data from the ECR and by giving permission for the retrospective registration of additional data. All hospitals voluntarily participated.

**Fig. 1:**
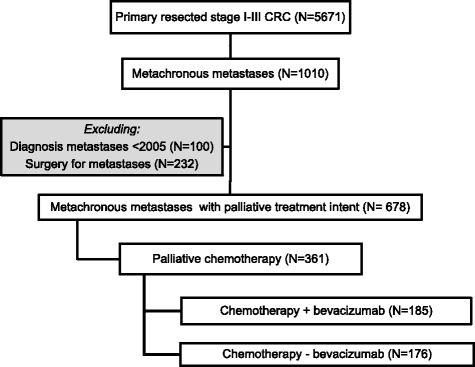
An overview on the palliative systemic treatment of metachronous metastases for patients diagnosed between 2003 and 2008 with stage I-III colorectal cancer in the south of the Netherlands

Metachronous metastases were defined as distant metastases of primary CRC in other organs, diagnosed at least 3 months after CRC diagnosis. However, the majority of metachronous metastases diagnoses (94 %) occurred at least 6 months after CRC diagnosis. Patterns of metastatic disease were determined based on the site of metastasis according to the International Classification of Diseases for Oncology (ICD-O), which could involve multiple localizations. Median time from primary diagnosis to data collection was 5.3 years (range 1.5–8.8 years). All consecutive patients with metachronous metastases from primary resected CRC were selected (*n* = 1010). Patients diagnosed with metachronous metastases before 2005 (*n* = 100) were excluded as bevacizumab is registered and recommended as a therapeutic option in addition to first-line chemotherapy in the Netherlands since 2005 [8]. Subsequently, patients undergoing surgery for metastases were excluded (*n* = 232), resulting in a study population treated with palliative intent (*n* = 678) of whom 361 received palliative chemotherapy (with or without palliative procedures; bypass, anastomosis, stoma). These latter patients were categorized into two treatment groups according to the prescription of bevacizumab in addition to palliative chemotherapy (Fig. 1). In the current study, we focused on the first-line palliative treatment as this is the indication for which bevacizumab is registered in the Netherlands.

### Statistical analysis

Variation in the prescription of bevacizumab between hospitals of diagnosis in the ECR-region was assessed using a χ^2^ test. Also, differences in patient and tumor characteristics and the prescription of bevacizumab between chemotherapeutic regimens were tested by means of a χ^2^ test. To discriminate independent predictors of treatment with bevacizumab, a multivariable logistic regression model was used. Adjustments were made for relevant patient and tumor characteristics: gender, age, comorbidity at time of CRC diagnosis, primary tumor localization, adjuvant chemotherapy, time to metastases, period of metastases diagnosis, number of metastases and the prescribed first-line chemotherapeutic regimen. In order to limit potential endogeneity bias due to the population-based nature of the data, a propensity score matched sample was created. Propensity scores were determined with a logistic regression model in which bevacizumab was the variable of interest and the independent variables were factors potentially associated with the use of bevacizumab (similar to variables taken into account in the multivariable logistic regression analysis). Patients were then matched within tight bounds of the propensity scores (probability could vary by no more than 1 %). Overall survival time was defined as the time from diagnosis of the first metachronous metastatic site to death or lost to follow-up. Patients still alive at the end of follow-up (January 1^st^, 2014) and those who emigrated were censored. Crude survival estimates according to the prescription of bevacizumab were calculated with the Kaplan-Meier method and presented up to 48 months in both the total study population and the propensity score matched sample. Median survival (MS) was presented in months and corresponding 95 % confidence intervals (CIs). A log-rank test was carried out to evaluate significant differences between survival curves. Multivariable Cox regression analyses were performed in both the total study population and propensity score matched sample to evaluate the independent effect of additional bevacizumab on the risk of death. Adjustments were made for the clinically relevant variables age, comorbidity, localization of primary tumor, adjuvant chemotherapy, time to metastases, period of metastases diagnosis, number of metastases, prescribed first-line chemotherapeutic regimen and the total number of systemic lines for the treatment of metastases. All analyses were performed with SAS/STAT® statistical software (SAS system 9.3; SAS institute, Cary, NC).

### Ethical considerations

In the Netherlands, the NCR and Dutch hospitals have a formal agreement that all cancer patients are informed about registration in the Cancer Registry and the possibility to decline registration. According to the Dutch law, all cancer patients are included in the NCR unless the patient has objected to be registered. Therefore, consent of the patient for this specific study was not applicable.

The NCR retrospectively collects data from medical records and is obligated to work according to laws in which the privacy of patients and doctors is fixed in regulations; the law about protection of privacy and the law “Geneeskundige BehandelOvereenkomst”. An independent Committee of Privacy reassures that the NCR works compliant to these regulations. In the Netherlands, retrospective studies with data collected from medical charts do not fall under the scope of the Medical Research Involving Human Subjects Act (‘Wet Medisch-wetenschappelijk Onderzoek”) as patient integrity is not violated in these studies. Therefore, this study was exempted from further medical ethics review.

## Results

**Fig. 2:**
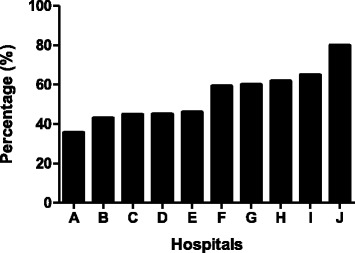
Proportion of patients receiving first-line palliative chemotherapy plus bevacizumab according to hospital of diagnosis (*n* = 361)

Out of 5671 primary resected stage I-III CRC patients diagnosed between 2003 and 2008, 1010 patients developed metachronous metastases (18 %). In total, 361 patients received first-line systemic therapy for the palliative treatment of metachronous colorectal metastases. Palliative procedures including a diverting stoma, bypass or anastomosis were performed in a minority of the patients (*n* = 18,5 %). Bevacizumab was prescribed in 51 % of the patients (*n* = 185), with proportions varying from 36 % to 80 % between the 10 hospitals in the ECR region (*p* < 0.0001, Fig. 2). An overview of patient and tumor characteristics according to the addition of bevacizumab to first-line systemic therapy is shown in Table 1.

**Table 1:** Patient and tumor characteristics according to the addition of bevacizumab to first-line systemic therapy (*n* = 361)

*N* = 361	Without bevacizumab (*n* = 176)		With bevacizumab (*n* = 185)			Combination chemotherapy (*n* = 219)		Single-agent chemotherapy (*n* = 142)		
	N	(%)	N	(%)	*P*-value	N	(%)	N	(%)	*P*-value
Gender					0.69					0.33
Male	75	(43)	72	(41)		126	(58)	89	(63)	
Female	101	(57)	113	(59)		93	(42)	53	(37)	
Age (years)					<0.0001					<0.0001
< 60	36	(20)	57	(31)		71	(32)	24	(17)	
60–75	91	(52)	111	(60)		128	(59)	72	(51)	
≥ 75	49	(28)	17	(9)		20	(9)	46	(32)	
Comorbidity					<0.001					<0.01
No	50	(28)	81	(44)		38	(28)	91	(42)	
1 comorbid condition	49	(28)	56	(30)		36	(26)	66	(30)	
≥ 2 comorbid conditions	63	(36)	33	(18)		52	(36)	44	(20)	
Unknown	14	(8)	15	(8)		14	(19)	18	(8)	
Primary tumor localization					0.13					
Rectum	68	(39)	85	(46)		107	(49)	47	(33)	<0.01
Colon	108	(61)	100	(54)		112	(51)	95	(67)	
Adjuvant chemotherapy					0.34					<0.01
No	103	(59)	100	(54)		137	(63)	67	(47)	
Yes	73	(41)	85	(46)		82	(37)	75	(53)	
Time to metastases (years)					0.12					0.59
< 1 year	57	(32)	43	(23)		57	(26)	42	(30)	
1–2 years	56	(32)	71	(38)		82	(37)	46	(32)	
≥ 2 years	63	(36)	71	(38)		80	(37)	54	(38)	
Period of diagnosis metastases					<0.01					0.81
2005–2006	70	(40)	41	(22)		69	(31)	42	(30)	
2007–2008	57	(32)	78	(42)		83	(38)	52	(37)	
2009–2011	49	(28)	66	(36)		67	(31)	48	(34)	
Number of organs affected					0.29					0.39
1 organ	72	(41)	85	(46)		92	(42)	65	(46)	
2 organs	69	(39)	59	(32)		83	(38)	44	(31)	
≥ 3 organs	35	(20)	41	(22)		44	(20)	33	(23)	
First-line chemotherapy					<0.0001					
Single agent chemotherapy	110	(63)	32	(27)						
Combination chemotherapy	66	(37)	153	(83)						
Bevacizumab										<0.0001
Yes						153	(70)	32	(23)	
No						66	(30)	110	(77)	

### Patient and tumor characteristics

Of the 361 patients treated with first-line systemic therapy, 219 patients received combination chemotherapy (CAPOX/FOLFOX in 96 %) and 142 patients received single-agent chemotherapy (capecitabine 74 %, irinotecan 20 %). Patient and tumor characteristics of patients treated with these chemotherapeutic regimens are shown in Table 1. Patients receiving combination chemotherapy were younger, had less comorbidities, were more often diagnosed with rectal tumors and less often received prior adjuvant chemotherapeutic treatment than patients receiving single-agent chemotherapy. Moreover, patients treated with combination chemotherapy more frequently received bevacizumab (*n* = 153, 70 %) than patients treated with single-agent chemotherapy (*n* = 32, 23 %, *p* < 0.0001).

### Predictors of treatment with bevacizumab

In multivariable regression analysis including adjustment for the type of prescribed first-line chemotherapeutic regimen, several factors were shown to influence the probability to receive additional first-line bevacizumab (Table 2). It was confirmed that patients treated with combination chemotherapy were more likely to receive bevacizumab than patients treated with single-agent chemotherapy (OR 9.666, 95 % CI 5.43–17.05). Moreover, the odds for treatment with bevacizumab was higher for patients diagnosed with metastases in a recent time period than patients diagnosed with metastases shortly after the introduction of bevacizumab in Dutch guidelines (2005–2006). The probability to receive bevacizumab was lower for patients with ≥2 comorbidities than patients without comorbidity (OR 0.4, 95 % CI 0.21–0.81) No association was observed between age and the use of bevacizumab. However, elderly patients (≥75 years) were less likely to receive combination chemotherapy (OR 0.2, 95 % CI 0.11–0.30).

**Table 2:** Proportion of patients treated with bevacizumab among patients who received chemotherapy, and predictors of treatment with bevacizumab in first line, adjusted for all factors listed (*n* = 361)

*N* = 361	N	(%)	OR	95 % CI
Gender				
Male	72	(49)	Ref	
Female	113	(53)	1.3	0.79–2.16
Age (years)				
< 60	57	(60)	Ref	
60–74	111	(56)	1.1	0.61–2.05
≥ 75	17	(26)	0.5	0.22–1.27
Comorbidity				
No	81	(62)	Ref	
1 comorbid condition	56	(54)	0.8	0.42–1.45
≥ 2 comorbid conditions	33	(34)	0.4	**0.21–0.81**
Unknown	15	(48)	0.7	0.27–1.65
Primary tumor localization				
Rectum	85	(55)	Ref	
Colon	100	(48)	0.9	0.37–2.26
Adjuvant chemotherapy				
No	100	(49)	Ref	
Yes	85	(54)	1.7	0.98–2.96
Time to metastases (years)				
< 1 year	43	(43)	Ref	
1–2 years	71	(55)	1.5	0.78–2.82
≥ 2 years	71	(53)	1.2	0.60–2.30
Period of diagnosis metastasis				
2005–2006	41	(37)	Ref	
2007–2008	78	(58)	3.0	**1.62–5.70**
2009–2011	66	(57)	3.3	**1.67–6.74**
Number of organs affected				
1 organ	85	(54)	Ref	
2 organs	59	(46)	0.5	**0.29–0.91**
≥ 3 organs	41	(53)	0.9	0.46–1.74
First-line chemotherapy				
Single agent chemotherapy	32	(23)	Ref	
Combination chemotherapy	153	(70)	9.6	**5.43–17.05**

N; number of patients receiving bevacizumab in the first-line of systemic treatment

%; percentage of patients receiving bevacizumab in the first-line of systemic treatment

*OR* odds ratio, *CI* confidence interval

Bold data; P-value <0.05

### Survival analysis

**Fig. 3:**
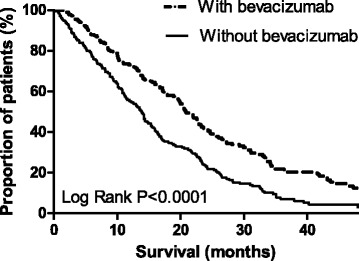
Overall survival according to the addition of bevacizumab to first-line systemic therapy (*n* = 361)

As shown in Fig. 3, the addition of bevacizumab to first-line palliative chemotherapy was associated with an improved median overall survival, from 14 months (95 % CI 11–16) to 22 months (95 % CI 19–24) (log rank *p* < 0.0001). In the propensity score matched sample, including 60 patients (with bevacizumab *n* = 30, without bevacizumab *n* = 30), comparable results were found with a median overall survival of 13 months (95%CI 7.62–18.92) versus 25 months (95%CI 7.62–18.92) (log rank *p* < 0.05). In multivariable analysis, the addition of bevacizumab to palliative chemotherapy resulted in a reduced hazard ratio on death, in both the total study population (HR 0.6, 95 % CI 0.45–0.73) and propensity score matched sample (HR 0.3; 95 % CI 0.14-.079, Table 3). After stratification for the prescribed first-line chemotherapeutic regimen, the beneficial effect of the addition of bevacizumab was observed in the subset of patients receiving combination chemotherapy (HR 0.6; 95 % CI 0.40–0.81), but not in patients treated with single-agent chemotherapy (HR 0.9, 95 % CI 0.60–1.54).

**Table 3:** Multivariable Cox regression analysis modelling the independent effect of additional bevacizumab on the risk of death, adjusted for all factors listed

	Total study population			Propensity score matched sample		
	(*N* = 361)			(*N* = 60)		
	HR	95 % CI	*P*-value	HR	95 % CI	*P*-value
Age (years)						
< 60	Ref			Ref		
60–74	1.0	0.76–1.38	0.86	1.8	1.82–4.13	0.15
≥ 75	1.3	0.87–1.90	0.21	0.8	0.20–3.53	0.80
Comorbidity						
No	Ref			Ref		
1 comorbid condition	0.9	0.69–1.22	0.56	1.1	0.40–2.86	0.88
≥ 2 comorbid conditions	0.8	0.54–1.04	0.08	1.0	0.34–3.01	1.07
Unknown	0.7	0.47–1.17	0.19	0.6	0.21–1.98	1.00
Primary tumor localization						
Rectum	Ref			Ref		
Colon	**1.3**	**1.01–1.65**	**<0.05**	0.6	0.21–1.98	0.35
Adjuvant chemotherapy						
No	Ref			Ref		
Yes	1.0	0.82–1.34	0.68	1.0	0.44–2.32	0.97
Time to metastases (years)						
< 1 year	Ref			Ref		
1–2 years	1.1	0.87–1.57	0.28	0.7	0.25–1.87	0.46
≥ 2 years	1.0	0.72–1.31	0.85	0.4	0.16–1.22	0.11
Period of diagnosis metastasis						
2005–2006	Ref			Ref		
2007–2008	1.1	0.81–1.64	0.42	2.3	0.64–8.34	0.20
2009–2011	1.1	0.78–1.71	0.47	2.5	0.67–9.62	0.16
Number of organs affected						
1 organ	Ref			Ref		
2 organs	1.2	0.96–1.63	0.19	1.8	0.74–4.78	0.18
≥ 3 organs	**1.6**	**1.12–2.21**	**<0.01**	**4.3**	**1.48–13.00**	**<0.01**
First-line chemotherapy						
Single agent chemotherapy	Ref			Ref		
Combination chemotherapy	0.9	0.69–1.27	0.69	**0.2**	**0.07–0.49**	**<0.001**
Additional bevacizumab						
No	Ref					
Yes	**0.6**	**0.49–0.89**	**<0.01**	**0.3**	**0.14–0.79**	**<0.05**
Number of systemic lines						
1 line	Ref					
2 lines	**0.6**	**0.44–0.84**	**<0.01**	0.5	0.16–1.74	0.29
≥ 3 lines	**0.4**	**0.31–0.58**	**<0.0001**	**0.3**	**0.13–0.72**	**<0.01**

*N* number of patients, *HR* hazard ratio, *CI* confidence interval

Bold data; P-value <0.05

## Discussion

To our knowledge, this is the first study providing population-based data on the use of bevacizumab in the metachronous setting, which has been suggested to differ from synchronous manifestation of disseminated disease with respect to tumor biology and prognostics [9–11]. Bevacizumab was prescribed in approximately half of the patients with metachronous metastases receiving first-line palliative treatment between 2005 and 2011 in the southern part of the Netherlands, achieving a median overall survival of 22 months. Prescription of bevacizumab varied significantly between hospitals of diagnosis and depended on the prescribed chemotherapeutic regimen.

The inter-hospital variation in the adoption of bevacizumab as observed in our study may reflect differences in policy and attitude towards the use of this anti-angiogenic agent in daily practice [12]. Bevacizumab was FDA-approved following the landmark publication by Hurwitz et al in which a survival benefit was demonstrated in patients treated with irinotecan, bolus fluorouracil and leucovorin (IFL) [6]. However, by the time bevacizumab was adopted in clinical practice, a shift towards oxaliplatin-based chemotherapy had taken place in the Netherlands [13–15]. Due to the initial absence of efficacy data in addition to these oxaliplatin containing regimens and the controversial results that were reported later on [16], the role of bevacizumab remained a highly debated topic. Also, the recent introduction of antibodies against epidermal growth factor receptors (EGFR) [17] strengthened the debate, as the question was raised which targeted agent should be preferred in the first-line systemic treatment [18]. In order to prevent an expanding gap between “believers” and “non-believers” in the current era of evolving treatment options for mCRC, ongoing efforts are needed to establish an evidence based opinion on the use of bevacizumab.

In line with the Dutch guidelines, the majority of patients with metachronous metastases received oxaliplatin-based chemotherapy (CAPOX or FOLFOX) whereas fewer patients were treated with single-agent chemotherapy (mostly capecitabine). Elderly patients and patients with multiple comorbidities were less often considered candidates for treatment with oxaliplatin-based chemotherapy, reflecting the generally accepted opinion that individual components of a systemic regimen should be selected on a number of factors, including patient related factors such as age, performance status and comorbidity [19]. Moreover, we observed that adjuvant chemotherapy influenced the choice of chemotherapy for the treatment of metachronous metastases. If adjuvant chemotherapy was prescribed, patients were less likely to receive combination chemotherapy as palliative treatment. This probably reflects the persistence of troublesome oxaliplatin induced polyneuropathy after adjuvant chemotherapy [20]. Since 2004, adjuvant oxaliplatin-based chemotherapy is considered the standard treatment schedule in the Netherlands for high risk stage II and stage III colonic tumors [21, 22]. For rectal tumors, however, adjuvant chemotherapy is generally not recommended, which probably explains the relatively higher proportion of oxaliplatin-based regimens for the treatment of metastases in this subset of patients.

The likelihood of treatment with bevacizumab was shown to depend strongly on the prescribed chemotherapeutic regimen for the metastatic disease. If a patient was considered a candidate for combination-chemotherapy, bevacizumab was prescribed in approximately 70 % of the cases. On the opposite, if single-agent chemotherapy was prescribed, only 23 % of the patients received bevacizumab. These findings are in line with results from observational cohort studies in the U.S. [13–15]. Of course, it could be speculated that bevacizumab was prescribed in combination with further lines of chemotherapy, as the results from the CAIRO III study revealed equal results for combined and sequential treatment chemotherapy strategies [23]. However, very few patients included in the current study received bevacizumab in further lines of treatment (data not shown).

In accordance with observations from the current literature in which age has been identified as one of the most important factors when deciding the type of therapy for patients with mCRC [24, 25], we observed that elderly patients (≥75 years) were less likely to receive combination-chemotherapy than younger patients. However, advanced age did not influence the probability to receive bevacizumab if adjustments were made for the prescribed chemotherapeutic regimen. Thus, age influenced primarily the choice of cytostatic backbone. This observation reflects the lack of data on the benefit-risk ratio of combination-chemotherapy regimens in older patients. It has been shown that the bevacizumab related adverse events do not increase with age, except for arterial thromboembolic events [26]. However, for this complication other patient related factors appeared to be stronger predictive factors than age [26]. Bevacizumab should therefore be considered a potential therapeutic option for elderly patients with mCRC and age alone should not be considered an absolute contraindication [27].

The addition of bevacizumab to first-line palliative therapy was associated with a median overall survival of 22 months, which is consistent with reports from observational studies on mCRC from the period 2002–2007 [13–15]. Of course, this observed improvement in overall survival with the addition of bevacizumab was biased by the prescription of more potential cytostatic backbone regimens in the presence of bevacizumab, and by patient selection by the treating physician. Nevertheless, after adjustment for important prognostic factors such as the prescribed chemotherapeutic regimen, a reduced hazard of death was observed in patients receiving additional bevacizumab. Moreover, results remained present in the propensity score matched sample, in which an effort is made to limit potential endogeneity bias. After stratification for the type of chemotherapy, the beneficial effect of additional bevacizumab achieved significance only in the subset of patients treated with combination-chemotherapy, probably because patient numbers were too small in the subset of single-agent backbone therapy. Although these non-randomized observational data should be interpreted with caution, together with the demonstrated benefit of bevacizumab across chemotherapy regimens in several RCTs [6, 16, 28–30] and observational studies [13–15], they strengthen the suggestion that bevacizumab is likely to add activity to various chemotherapy regimens with which it is combined.

Despite the accurate and concise patient registration, use of the ECR also implies limitations to our data. No data on relevant prognostic factors such as extent of metastatic burden were available. Moreover, data on patient and tumor characteristics such as comorbidity are registered by registration personnel approximately 6–9 months after primary tumor diagnosis. Therefore, it is not possible to provide data on the specific comorbidities present at the time of treatment for metachronous metastases.

## Conclusions

In conclusion, in this population-based study it was revealed that addition of bevacizumab to the first line treatment of metachronous metastases of CRC is likely to be an independent beneficial factor for overall survival in patients receiving oxaliplatin containing chemotherapy. Moreover, a significant inter-hospital difference in the prescription of bevacizumab was found, reflecting differences in attitude towards and policies in the use of bevacizumab in clinical practice. Ongoing efforts should be made to further define the position of targeted agents in the treatment of metachronous metastases from CRC.

## Abbreviations

CI: confidence interval
CRC: colorectal carcinoma
ECR: Eindhoven cancer registry
HR: hazard ratio
mCRC: metastatic colorectal carcinoma
